# Interventions Aiming to Improve Breastfeeding Duration Among Primiparous Women: A Scoping Review

**DOI:** 10.3390/pediatric18020035

**Published:** 2026-03-03

**Authors:** Jasmine Keurentjes, Laurie-Eve Brault, Stéphanie Bégin, Maude Perreault, Véronique Gingras

**Affiliations:** 1 Nutrition Department, Université de Montréal, Montreal, QC H3T 1A8, Canadamaude.perreault@umontreal.ca (M.P.); 2 Research Center of the Centre Hospitalier Universitaire Sainte-Justine, Montreal, QC H3T 1C5, Canada; stephanie.begin.hsj@ssss.gouv.qc.ca; 3 Centre Jean-Jacques Gauthier, Research Center of the Centre Intégré Universitaire de Santé et de Services Sociaux du Nord-de-l’Île-de-Montréal, Montreal, QC H2M 1P2, Canada

**Keywords:** breastfeeding, lactation, duration, interventions, prenatal, postnatal, primiparous, nulliparous, mothers

## Abstract

**Background:** Worldwide breastfeeding initiation and exclusive rates at 6 months remain lower than recommended. Our scoping review aimed to identify interventions to improve breastfeeding duration in primiparous women. We assessed interventions’ effectiveness during the prenatal and postnatal periods separately or combined. **Methods:** Eight databases and grey literature were searched in March 2023, using a keyword search strategy. **Results:** We identified 16,161 articles from 2013 to 2023, and 35 met our eligibility criteria. The studies were conducted mostly in low–middle income countries (62.9%), and they proposed a variety of interventions in the prenatal period (n = 8), the postnatal period (n = 11) and in a combination of both periods (n = 16). It appears that a combination of various interventions, in both the prenatal and postnatal periods, targeting young women who intended to breastfeed, with low education levels, and with a partner, showed positive effects on exclusive breastfeeding rates until 6 months. Combined approaches such as workshops or individual education and support sessions during the prenatal period with support by professionals or peers until at least 6 months also showed improvements on breastfeeding duration. **Conclusions:** Our scoping review was the first to have identified potentially effective interventions, alone or in combination, to improve breastfeeding duration among primiparous women. Further studies should be conducted to cover a longer period, beyond six months. They should also explore the role of sociodemographic factors, such as ethnicity, in interventions’ effects.

## 1. Introduction

Since 2003, the World Health Organization (WHO) and the United Nations International Children’s Emergency Fund (UNICEF) recommend exclusive breastfeeding from birth until 6 months of age, and continued breastfeeding combined with complementary foods for the following two years or as long as desired [1]. According to WHO, exclusive breastfeeding (EBF) is defined as feeding the baby only breast milk, i.e., “no other liquids or solids are given—not even water—with the exception of an oral rehydration solution, or drops/syrups of vitamins, minerals or medicines” [2]. Breastfeeding is the most complete, sustainable and recommended way to nourish a baby due to its nutritional and immunological benefits, among other factors [3]. The longer breastfeeding lasts, the lower are the risks of developing infections and chronic diseases, and the greater the protection is for both babies and mothers [4]. Among its benefits, breastfeeding reduces the risks of developing obesity and diabetes for babies, as well as ovarian or breast cancer for mothers [5,6,7,8]. These benefits explain, in part, the recommendation to breastfeed for up to 2 years of age.

Worldwide exclusive breastfeeding rates for infants under six months of age are 48% [9]. At one year, 71% of mothers worldwide breastfeed their child and 45% of them still do at 2 years [9]. Several studies have attempted to better understand these low rates of exclusive breastfeeding at 6 months of age, including which factors are more predictive of a shorter breastfeeding duration. Among them, a lower income and education level have been associated with lower breastfeeding duration [10,11]. Younger women with less breastfeeding experience, including primiparous or multiparous mothers without previous lactation experience, were also more likely to cease breastfeeding early [11,12].

Breastfeeding duration is also related to a positive prior breastfeeding experience [13,14]. Many studies suggested that breastfeeding education and support are important as they may increase the likelihood of initiation and breastfeeding duration [10]. Indeed, evidence highlights the need for early interventions to enhance primiparous women’s experiences and perceptions [13,14]. Numerous international organizations exist such as The Global Breastfeeding Collective (“a partnership of prominent international agencies calling on donors, policy makers and civil society o increase investment in breastfeeding worldwide work to improve breastfeeding duration”) [9]. They have identified recommendations to protect, promote and support breastfeeding, and, among them, there is the “improvement of accessible skilled breastfeeding counselling” [9]. Many studies tested the effectiveness of interventions and programs to promote breastfeeding initiation and duration (e.g., peer counselling and multidisciplinary professionals’ support at home, in hospital or by phone) during the prenatal and/or postnatal periods [15,16,17,18]. However, not many studies targeted primiparous women specifically, although evidence suggests they are more likely to discontinue breastfeeding early [13]. In fact, many factors can influence exclusive breastfeeding among primiparous mothers and may help identify other important variables to consider when designing interventions for this population. In the scoping review by Kusvitasari et al., the determinants identified were as follows: education, income, peer support and others psychosocial factors such as favorable intention or motivation, self-confidence and level of knowledge about breastfeeding. These factors can positively influence exclusive breastfeeding when mothers are well-prepared and supported during lactation [19].

To our knowledge, only one review (n = 9 articles) looked at multiple interventions to promote EBF and included a majority of young mothers or primiparous women, but only in high income country, which limits generalization of the results [15]. Other reviews or meta-analyses looked at interventions to promote breastfeeding duration, but they did not focus exclusively on primiparous parents [18,20]. No review has made an overview of the most effective actions to support primiparous mothers to breastfeed longer, as recommended by the WHO and UNICEF.

This scoping review aims to identify effective interventions to improve breastfeeding duration in primiparous women by the following: (1) assessing interventions’ effectiveness during (1a) the prenatal period, (1b) the postnatal period or (1c) combined periods; and (2) exploring the role of sociodemographic characteristics of primiparous mothers and their families.

## 2. Materials and Methods

This scoping review has been preregistered in Open Science framework (OSF; https://osf.io/h8t2j; accessed on 25 January 2026). The Preferred Reporting Items for Systematic reviews and Meta-Analyses Extension for Scoping Reviews guidelines (PRISMA-ScR) and updated methodological guidance for the conduct of scoping reviews were followed [21,22]. No ethics clearance was required due to the nature of the work.

### 2.1. Types of Sources of Evidence and Search Strategy

We performed a systematic keyword literature search strategy on the following electronic databases: MEDLINE, Embase, CINAHL, Cochrane, Web of Science, Cab Abstract, Sociological abstract and Social Science abstract. We also searched the grey literature (ProQuest Dissertations, Theses Global and Google Scholar); yet, although grey literature was considered in the search strategy, no grey literature sources were ultimately included based on our inclusion criteria. The search strategy was developed in partnership with a research librarian at the Université de Montréal [See Table S1]. We included studies published between 2013 and 2023 (randomized or non-randomized controlled interventions trials) given the large number of studies in the literature.

### 2.2. Eligibility Criteria

Articles published in English or in French that tested interventions to improve breastfeeding duration until at least 3 months were included, focusing on those with effects observed beyond initiation. Eligibility criteria also included studies with primiparous or multiparous women with no previous breastfeeding experience and ≥18 years old. We excluded studies including women with twin pregnancies, premature birth or multiparous women as this could impact the effectiveness of the interventions. Public health interventions or interventions not specifically targeting women were not eligible. Lastly, we excluded studies from countries who do not adhere to the WHO and UNICEF breastfeeding recommendations.

### 2.3. Screening Process

Articles were imported into Covidence (Covidence systematic review software, Veritas Health Innovation, Melbourne, Australia. Available at www.covidence.org, accessed in 12 September 2024). Duplicates were automatically removed. All articles were first screened by titles and abstracts, and those selected were then screened by a full text read based on eligibility criteria. Studies identified by our search were screened independently by at least two reviewers (JK, SB and LEB) according to eligibility criteria. Any conflict was resolved by consensus or by a fourth reviewer (VG). We did not include full-text articles that were not accessible to the research team.

### 2.4. Quality Assessment

The revised Cochrane risk of bias tool for randomized studies (ROB2)—and in non-randomized studies—of Interventions (ROBINS-1) templates were used to assess the quality of the articles by two reviewers independently (JK and SB) [23,24]. Any disagreements were resolved by consensus with the research team or by a third reviewer (VG). In ROB-2 tool (RCT), the categories were as follows: “randomisation process, timing of identification or recruitment of participants, deviations from intended, measurement of the outcome intervention and the selection of the reported result” [23]. For ROBINS-1 tool (nonrandomized studies of interventions), the categories were similar as in the RoB2 tool, except for the question about randomization that read as follows: “Bias due to confounding, selection of participants and classification of interventions”. Studies were summarized and classified into three levels: low risk, some concerns and high risk of bias to make a critical appraisal of selected articles.

### 2.5. Data Extraction and Synthesis

One reviewer (JK) extracted relevant information using a predefined Excel table. Another reviewer (SB) validated the extracted information. Any conflicts were discussed and resolved by consensus with the research team. The predefined extraction table was inspired by Peters et al., 2021 and included the following studies’ characteristics: title, authors, type of study, year of publication, countries where the study was conducted and the sample size [21]. The extraction table also included a brief description of the interventions as well as breastfeeding outcome measures (duration until 12–24 months when applicable) including the effect size with corresponding 95% confidence interval (CI), *p* value, odds ratio (OR) and relative risk (RR), funding sources, conflicts of interest (if applicable) and the quality assessment of the included articles. Data were separated into three time periods: prenatal, postnatal or combination of the two periods. Participants’ sociodemographic characteristics were also extracted, including the following information: mean age, income and education levels, intention to breastfeed, marital status and ethnicity.

### 2.6. Statistical Models

Considering the heterogeneity of the available data, a meta-analysis was not conducted.

## 3. Results

### 3.1. Study Selection

**Figure 1:**
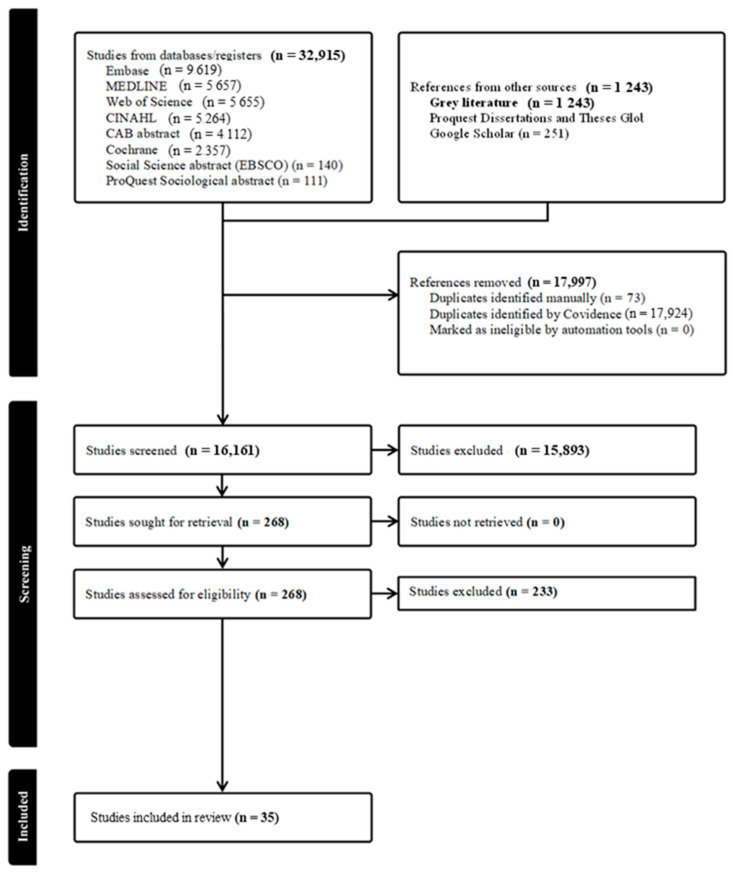
Preferred Reporting Items for Systematic reviews and Meta-Analysis (PRISMA) flow diagram for our scoping review.

The Preferred Reporting Items for Systematic reviews and Meta-Analysis (PRISMA) flow diagram is presented in Figure 1 below. A total of 34,158 articles were imported for screening (n = 32,915 from databases and n = 1243 from grey literature), 17,924 were duplicated and automatically managed by Covidence and 73 were manually identified as duplicates. The first screening by titles and abstracts excluded 16,161 articles, and the second screening by full-text articles excluded 268 articles. A total of 35 eligible articles were retained (n = 7175 mother participants). Most of the studies excluded involved multiparous women (n = 124) [See PRISMA flow diagram (Figure 1)].

### 3.2. Study Characteristics

#### 3.2.1. Study Design

Most studies were randomized controlled trials (RCTs) (n = 28), quasi-experimental intervention studies (n = 6) and non-randomized controlled trials (n = 1). Articles proposed a variety of interventions in the prenatal period (n = 8), the postnatal period (n = 11) and in a combination of both periods (n = 16). Several studies included standard of care in both the control group (CG) and intervention group (IG), sometimes extending into the postpartum period. As the aim is to analyze the impact of interventions that are not already implemented or offered as standard, the classification by the period was based on the specific intervention(s) being tested.

#### 3.2.2. Country of Studies

**Figure 2:**
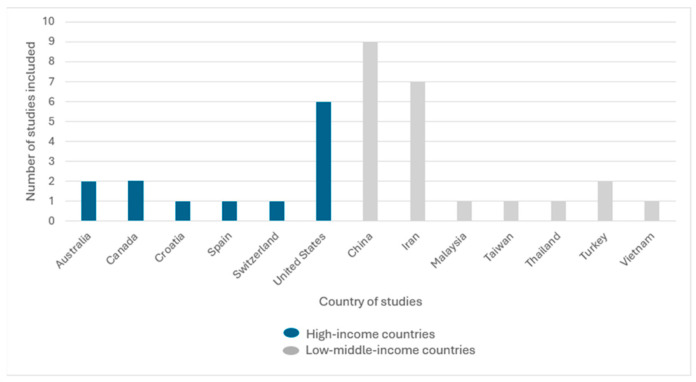
Number of studies included by country of studies.

Studies were conducted in a variety of countries around the world (Figure 2). Most of them were in low–middle-income countries (n = 22) and in high-income countries (n = 13) [25].

#### 3.2.3. Population Characteristics

In general, participants were primiparous women with a mean age of 27 years old and mostly living with a partner. Many studies (n = 12) included the partners in their intervention. There was a great variability in terms of income and education levels with a trend towards collegial or university-level education. In 19 articles, mothers reported having the intention to breastfeed, while other studies did not report on breastfeeding intentions. Ethnicity was heterogenous across studies. The words mother and father are employed in this review according to the terminology used in the analyzed studies.

#### 3.2.4. Quality Assessment of Included Studies

Most studies presented some concerns or a high risk of bias (n = 19 RCT, nonrandomized or quasi-experimental studies) given potential issues or significant bias in one or more of the categories evaluated in the quality assessment tools [See Tables S2 and S3] [24].

## 4. Interventions During the Prenatal Period and Breastfeeding Duration

A total of eight studies examined the effect of interventions during the prenatal period on breastfeeding duration (Table 1). Among them, six were RCTs (one pilot study), and two were quasi-experimental studies. The interventions tested in the prenatal period were group interventions (n = 4), individual interventions (n = 3) and online intervention (n = 1).

### 4.1. Group Interventions During the Prenatal Period

Many studies proposed group interventions [26,27], with some also including the partner [28,29]. In these studies, mothers receiving an educational breastfeeding training session had a significantly increased rate of EBF at 6 months [26,28,29]. In the study by Tseng et al., three workshops between 34 and 35 weeks of pregnancy covering theoretical and practical elements of breastfeeding, with a breastfeeding educator, increased breastfeeding rates until 6 months as compared to standard of care [29]. Similar findings were shown by Su et al. using a quasi-experimental design with only one prenatal session [28]. Both interventions included mothers and fathers and covered aspects such as discussion about their respective roles, expectations, etc. Another study from Ansari et al. proposed an intervention that included educational sessions in groups led by midwives, a handout for mothers and access for mothers to a phone support line as needed [26]. They found greater EBF rates until 6 months in the intervention compared to the control group. Finally, Naroee et al. showed that four motivational group education sessions with mothers between their 32nd and 34th weeks of pregnancy also contributed to maintaining EBF until 4 months postpartum [27]. Despite the small sample sizes in these studies and the diverse quality and designs, prenatal group sessions seem to positively influence EBF duration.

**Table 1:** Interventions during the prenatal period to improve breastfeeding duration ^1^.

Authors, Publication Date	Research Design; Study Location	Sociodemographic Characteristics	Sample Size, Interventions	Findings on Breastfeeding Duration	Funding Sources	Conflicts of Interest	Quality Assessment ^2^
Ansari et al., 2014 [26]	Randomized controlled trial; Iran (Ahvaz)	Mean age: 26.6 ± 5.5 Education level: IG: Secondary (31.5%); CG: Diploma (non-specified) (46.7%) Income level: Between 180 and 455$ in majority (Every $ was equal to 1100 Tumans in 2014) Intention to breastfeed: NE Marital status: Married (IG: 66%; CG: 70%) Ethnicity: NE	IG: (n = 60) Standard care + Breastfeeding education sessions (2 × 2 h classes, 2 days apart) in group by a midwife and a breastfeeding coach during prenatal period + Support phone calls as needed + Breastfeeding guide CG: (n = 60) Standard care	EBF at 6 months: IG: 73.3% CG: 26.6% (*p* < 0.001)	University of Medical Sciences	None declared	−
Demirci et al., 2022 [30]	Randomized controlled trial (pilot study); United States (Pittsburgh)	Mean age: IG: 31.5 ± 4.5; CG: 31 ± 7.5 Education level: Attended university (Bachelor’s degree) (50%) Income level: NE (Employed at enrollment in majority) Intention to breastfeed: Yes Marital status: Married (IG: 78%; CG: 72%) Ethnicity: White (IG: 94%; CG: 83%) dd	IG: (n = 18) Antenatal milk expression training sessions with in-person sessions and video demonstrations on milk expression techniques weekly by an IBCLC + Milk expression by mothers individually (1–2 x/week) CG *: (n = 16) Educational breastfeeding documents provided weekly by researchers * IG and CG took place between 37 and 40 weeks of pregnancy (4 follow-ups)	EBF at 3–4 months *: IG: 78.0% CG: 69.0% * No information on significance of the results	American Nurses Foundation and Central Research Development Fund of Pittsburgh University	None declared	++
Naroee et al., 2020 [27]	Quasi-experimental design; Iran (Zahedan)	Mean age: IG: 22.4 ± 3.9; CG: 23.4 ± 5.0 Education level: High school diploma (IG: 34.3%; CG: 41.4%) Income level: >300 IRR (IG: 81.4%; CG: 74.3%) Intention to breastfeed: NE Marital status: Married or with a partner in majority Ethnicity: Baluch or Fars nationality in majority	IG: (n = 68) Standard care + 4 motivational group education sessions (45–60 min, 2 x/week) from 32 to 34 weeks of pregnancy CG: (n = 69) Standard care	Number of days of exclusive breastfeeding: IG: 137.68 ± 65.5 CG: 99.5 ± 80.6 (*p* = 0.003)	Office of Vice-President for Research and Information technology in Zahedan University of Medical Sciences.	None declared	++
Su et al., 2016 [28]	Quasi-experimental design; China (Wuhan)	Mean age: Mothers: IG: 28 ± 4.2; CG: 29 ± 2.9; Fathers: IG: 30 ± 4.7; CG: 31 ± 3.2 Education level: College or university (IG: 77.8%; CG: 66.7%) Income level: >3000 ¥ (IG: 52.8%; CG: 63.9%) Intention to breastfeed: NE Marital status: With a partner in majority Ethnicity: NE	IG: (n = 36 couples) One prenatal workshop in group with the partner (60–90 min) led by the researcher (nurse) + Breastfeeding brochure CG (n = 36 mothers): Same as IG without the partners (Certified with Baby-Friendly hospital Initiative)	EBF at 6 months: IG: 40.0% GC: 17.6% (*p* = 0.04)	None reported	None declared	++
Taheri et al., 2022 [31]	Randomized controlled trial; Iran (Babol)	Mean age: 26 ± 4.9 Education level: Attended university (IG: 55.6%; CG: 69.4%) Income level: Sufficiency of Income for expenses to some extent according to the authors (IG: 69.4%; CG: 63.9%) Intention to breastfeed: NE Marital status: With a partner in majority Ethnicity: NE	IG: (n = 34) Six virtual educational PDF via Telegram application from 32 to 37 weeks of pregnancy CG: (n = 35) Standard care	EBF at 6 months: IG: 81.8% CG: 57.1% (*p* = 0.03)	BABOL University of Medical Sciences Grant	None declared	−
Tseng et al., 2020 [29]	Single-blinded, randomized controlled trial; Taiwan (Taipei)	Mean age: 33.1 ± 4.2 Education level: College or above (86%) Income level: NE Intention to breastfeed: Yes (IG: 35.5% CG: 34.0%) Marital status: Married (94.6%) Ethnicity: NE	IG: (n = 50) Standard care (Breastfeeding group class available if needed) + 3 Breastfeeding workshops with the partner (90 min) at 34, 35 and 36 weeks of pregnancy by a breastfeeding educator CG: (n = 43) Standard care (Certified with Baby-Friendly hospital Initiative)	EBF at 6 months: IG: 32.0% CG: 13.9% OR 2.82, 95% CI [1.0–8.1] (*p* < 0.05)	Ministry of Science and Technology in Taiwan	None declared	−
Wong et al., 2014 [32]	Randomized controlled trial; China (Hong Kong)	Mean age: 31.4 ± 4.3 Education level: Attended university or above (43%) Income level: (HK$) 30,000 or above (56.3%) Intention to breastfeed: Yes (78.3%) Marital status: With a partner in majority Ethnicity: NE	IG: (n = 233) Standard care (breastfeeding group class during prenatal period + Breastfeeding support available PP if needed) + 1 individual breastfeeding educational and support session (30–45 min) by a nurse during prenatal period + Breastfeeding brochure CG: (n = 236) Standard care	EBF at 6 months: IG: 14.6% CG: 12.7% (*p* = 0.55)	University of Hong Kong Grant	None declared	−
Zhao et al., 2021 [33]	Longitudinal single-blinded, randomized, parallel-group trial; China (Shanghai)	Mean age: IG: 30.8 ± 3.5 CG: 30.2 ± 3.2 Education level: College (IG: 50% CG: 56%) Income level: ≥10,000 ¥/months (IG: 64.3%; CG: 60.7%) Intention to breastfeed: NE Marital status: Married Ethnicity: NE	IG: (n = 84) Standard care + 4 individual education sessions including 1 on breastfeeding workshop before delivery date by an IBCLC (70 min) with partner + Support phone call by researchers (nurse and IBCLC) if needed CG: (n = 84) Standard care	EBF at 6 months: IG: 50.0% CG: 50.0% (*p* = 0.02)	Fudan university Nursing Research Funding	None declared	−

^1^ IG: Intervention group; CG: Control group; PP: Postpartum period; EBF: Exclusive breastfeeding; NE: not evaluated. ^2^ − Low risk of bias; ++ High risk of bias.

### 4.2. Individual Interventions During the Prenatal Period

Three studies tested individual educational and support breastfeeding interventions in the prenatal period [30,32,33]. Only Zhao et al. found a positive and significant impact on EBF until 4 months following an intervention that included an International Board-Certified Lactation Consultant (IBCLC)-led workshop before the mothers’ expected delivery date, to which fathers were encouraged to attend. Mothers could also phone the research team for any questions during their pregnancy [33]. Wong et al. found no effect on EBF rates of one individual educational and support session led by a registered nurse (with a brochure) [32]. A small pilot study in which four antenatal milk expression training sessions were offered between the 37th and 40th weeks of pregnancy, with milk expression 1–2 times per weeks at home, showed promising benefits on breastfeeding rates at 3–4 months postpartum but did not conclude on the significance of the results [30].

### 4.3. Online Interventions During the Prenatal Period

Only one study evaluated an online intervention to improve BF rate in the prenatal period. Taheri et al. evaluated the effect of using an application named Telegram which consists of six online PDF documents about breastfeeding [31]. They found a significant improvement in EBF at 6 months in the IG when compared to the CG. Breastfeeding content was similar to the other studies [27,28,29,31,33], but without the motivational and discussion part.

## 5. Interventions During the Postnatal Period and Duration of Breastfeeding

Eleven studies out of thirty-four examined interventions during the postnatal period (Table 2). Among them, nine were RCTs, and one was a pilot RCT study. Of those, five were individual interventions, three were remote interventions including phone calls and online platforms and three were a combination of interventions.

### 5.1. Individual Interventions During the Postnatal Period

#### 5.1.1. Face-to-Face Educational and Support Interventions

Three different studies evaluated support interventions with or without a partner, led by either a physician, a midwife or a nurse. Panahi et al. proposed midwife-led support training sessions for the mothers and their partners in the second and third weeks postpartum, and this intervention was associated with a significant increase in EBF duration in the IG when compared to the CG [34]. On the other hand, Abbott et al. looked at an early breastfeeding support intervention consisting of follow-ups at 2–3 weeks postpartum compared to follow-up at 6–8 weeks postpartum, which were led by a physician, a nurse or a midwife, and no significant effect on EBF was found [35]. Yin et al. evaluated an intervention in which the baby-led self-attachment breastfeeding technique support was offered, with the support of a nurse before hospital discharge (no details regarding the number of visits or time spent with the nurse were provided), and significant improvements in EBF duration at 6 months were found [36].

#### 5.1.2. Face-to-Face Interventions Including Education and Distribution of Breastfeeding Materials During the Postnatal Period

In a small pilot study with a low-income population where the intervention consisted of the loan of manual breast pumps to mothers and support offered during hospitalizations, no significant effect on EBF rate at 3 months postpartum was found [37]. Hermanson et al. examined the impact on breastfeeding duration of offering a pacifier to the baby from the first day of life compared to avoiding it during the first two weeks [38]. No differences in EBF rates at 4 and 6 months were found between the two groups [38].

### 5.2. Remote Interventions with Phone Calls and Online Platforms During the Postnatal Period

Chehreh et al. found significant improvements for EBF at 3 months in the IG when compared to the CG with a weekly motivational counselling phone call by peers for 3 months [39]. Forster et al. also proposed peer support through telephone calls for the first 6 months and found that they improved significantly the non-exclusive breastfeeding rate at 6 months after adjusting for breastfeeding intention, formulas given, site and hazard ratio [40]. Support by phone call at 2 weeks postpartum, then by e-mail at 1 and 3 weeks and with online material for both parents (Website, videos, coparenting booklet) was also studied in Abbass-Dick et al. [37]. No significant effect was found at 3 months postpartum for the breastfeeding duration in the IG when compared to the CG.

### 5.3. Combination of Interventions During the Postnatal Period

Gu et al. showed that individual support combined with educational workshops in groups with the partner within the first 6 weeks, complemented by support phone calls until 6 months postpartum, had a significant impact on EBF rates at 6 months [41]. Chegeni et al. showed significant results for EBF rates at 4 months in both IG when compared to the CG with support by phone calls or via a Messenger discussion group supervised by a nurse, and online learning material (e.g., video, educational pictures) [42]. Shariat et al. looked at educational breastfeeding training sessions with an audio package and brochures, but no information was given regarding whether it was offered in individual or in group settings. Regardless, this study found a significant impact on breastfeeding duration at 6 months and at 2 years [43].

**Table 2:** Interventions during the postnatal period to improve breastfeeding duration ^1^.

Authors, Publication Date	Research Design; Study Location	Sociodemographic Characteristics	Sample Size, Interventions	Findings on Breastfeeding Duration	Funding Sources	Conflicts of Interest	Quality Assessment ^2^
Abbass-Dick et al., 2015 [44]	Randomized controlled trial; Canada (Toronto)	Mean age: IG: 30.4 ± 3.7; CG: 30.7 ± 3.8 Education level: Attended university (IG and CG = 62.5%) Income level: >60,000 $ (IG: 81.3%; CG: 72%) Intention to breastfeed: Yes Marital status: Married (IG: 91.6%; CG: 87.9%) Ethnicity: Born outside of Canada (IG: 65.4%; CG: 72.5%)	IG: (n = 105 mothers) Standard care (Breastfeeding support in hospital and in the community) + Breastfeeding support provided face-to-face with the partner PP at hospital (discussion) with a lactation specialist (15 min) + Follow-ups by phone calls at 2 weeks and by e-mails at 1–3 weeks PP and through guide, videos, website, and/or e-mails) CG: (n = 104 mothers) Standard care	EBF at 3 months: IG: 67.3% CG: 60.0% (*p* = 0.27) Any breastfeeding at 3 months: IG: 96.2% CG: 87.6% (*p* = 0.02)	Canadian Institutes of Health Research/Canada Research Chair Program	None declared	−
Abbott et al., 2019 [35]	Randomized controlled trial; United States (Washington)	Mean age: IG: 25 ± 4.3; CG: 25.3 ± 4.5 Education level: College or more (IG: 56.3% CG: 60.8%) Income level: 25,000 to 40,000 $ (IG: 20.1%; CG: 20.5%) or not stated (IG: 34.3%; CG: 29.8%) Intention to breastfeed: Yes (IG: 66.9%; CG: 68.9%) Marital status: Married Ethnicity: White (IG: 58.6%; CG: 60.2%), Black (IG: 12.4%; CG: 9.4%), also Hispanic, Asian, Native American and others	IG: (n = 130) Early breastfeeding follow-ups (2–3 weeks PP) CG: (n = 135) Traditional breastfeeding follow-ups * (6–8 weeks PP) * With a midwife, nurse practitioner or physician at medical center (Certified with Baby-Friendly hospital Initiative)	EBF at 6 months: IG: 57.7% CG: 59.3% (*p* = 0.80) RR 0.97, 95% CI [0.79–1.19] Adjusted RR at 5–6 months 1.45, 95% CI [0.71–2.95]	Department of Clinical Investigation and Madigan Army Medical Center	None declared	+
Chegeni et al., 2022 [42]	Randomized controlled trial; Iran (Khorramabad)	Mean age: IG 1: 24.2 ± 5.2; IG 2: 23.6 ± 5.6; CG: 24.2 ± 5.6 Education level: Women: Housewife (under diploma or diploma non-specified); Husband: Nongovernmental job (diploma non-specified) in majority Income level: NE Intention to breastfeed: NE Marital status: Married in majority Ethnicity: NE	IG1 (n = 84): Standard care (education session at the hospital) + Educational package content validated by professionals + Follow-up phone calls by a nurse (Days 1,3,5 and 6 after hospital discharge PP) IG2 (n = 83): Same as IG 1, but support PP via text messages with an online mothers group supervised by a nurse CG (n = 82): Standard care	EBF at 4 months: IG 1: 77.4% IG 2: 79.5% GG: 44.8% (*p* < 0.001)	None reported	None declared	−
Chehreh et al., 2021 [39]	Single-blind randomized clinical trial; Iran (Ilam)	Mean age: IG: 25.9 ± 4.7; CG: 25.7 ± 5.2 Education level: ± diploma non-specified (IG: 51.7%; CG: 60%) or attended university (IG: 37.1%; CG: 34.7%) Income level: NE Intention to breastfeed: NE Marital status: NE Ethnicity: Kurdish (IG: 78.8%; CG: 93.7%)	IG (n = 95): Weekly breastfeeding motivational counseling phone calls with peers during 3 months PP (First call: 48 h post hospital discharge) + In-person follow-ups (debugging sessions) with peers and researchers (midwives, nurses) every 2 weeks until 12 weeks PP, if needed CG (n = 89): Standard care	EBF at 3 months: IG: 96.8% CG: 62.9% (*p* = 0.001)	None reported	None declared	+
Forster et al., 2019 [40]	Multicenter, unblinded, randomized controlled trial; Australia (Victoria)	Mean age: IG: 31.0 ± 5; CG: 31.2 ± 4.7 Education level: University degree or more (IG: 64%; CG: 70%) Income level: ($ AUD): Between 1000 and 2000$ or more (IG: 70%; CG: 71%) Intention to breastfeed: Yes Marital status: Married in majority Ethnicity: Born in Australia (IG: 48%; CG: 42%)	IG: (n = 501) Standard care (Access to a lactation consultant if needed at the hospital or 1–2 home visits PP by a midwife during week 1 + Participation in the program Maternal and Child Health Nurse if needed) + Ringing Up about breastfeeding—RUBY intervention: Support phone calls from peers until 6 months PP: (1) 24–48 h; (2) Days 4–6 every week for 12 weeks; (3) Every 3–4 weeks between week 12 and 6 months CG: (n = 515) Standard care	EBF at 6 months (may include foods or other liquids): IG: 54.0% CG: 48.0% Adjusted RR 1.10, 95% CI [1.02–1.18]	PhD scholarship from University of La Trobe	None declared	−
Gu et al., 2016 [41]	Prospective randomized controlled trial; China (Shanghai)	Mean age: IG: 29.6; CG: 29 Education level: Attended university (IG: 75.8%; CG: 66.4%) Income level: Between 100,000 and 300,000 ¥ (IG: 84.7%; CG: 83.6%) Intention to breastfeed: NE Marital status: Married in majority Ethnicity: Chinese women (According to inclusion criteria)	IG: (n = 157) Standard care (Breastfeeding education training session during prenatal period and PP + PP breastfeeding support by a nurse) + Theory of planned behavior’s program with nurse including: Individual support (Day 2–3 at hospital) + Educational group workshops in with the partner at hospital (30 min–1 h) (Day 2 + Week 6 PP) + Support phone calls: (1) Weeks 1–2 at 2x/week; (2) Week 3–6 at 1x/week; (3) Week 6 to Month 3 at 2x/months; (4) Months 3–6 according to work return CG: (n = 128) Standard care (Certified with Baby-Friendly hospital Initiative)	EBF at 4 months: IG: 56.7% CG: 15.6% (*p* < 0.001) EBF at 6 months: IG: 42.0% CG: 10.02% (*p* < 0.001)	Shanghai Science and Technology Committee grant	None declared	+
Hermanson et al., 2020 [38]	Prospective randomized controlled trial with parallel group design; Switzerland (Linköping)	Mean age: IG: 29.3; CG: 28.6 Education level: Attended university (IG: 81.6%; CG: 77.1%) Income level: NE Intention to breastfeed: Yes Marital status: Majority cohabiting with the father Ethnicity: Born in Switzerland (IG: 94.2%; CG: 89.8%)	IG: (n = 109) Recommendation of early pacifier use from the first day PP CG: (n = 100) Recommendation to avoid pacifier use for the first 2 weeks PP	EBF at 4 months: IG: 68.8% CG: 66.0% (*p* = 0.208) EBF at 6 months (Babies may have received small amounts of solid food): IG: 35.8% CG: 36.0% (*p* = 0.918)	None reported	None declared	+
Hoyt-Austin et al., 2023 [37]	Pilot randomized controlled trial; United States (California)	Mean age: 24 ± 5 Education level: Some college or associate’s degree or more (IG: 54%; CG: 52%) Income level: Low (Medicaid insurance in majority) Intention to breastfeed: NE Marital status: Married or live-in partner (IG + CG: 85%) Ethnicity: White (56%), Hispanic (36%) and Black (15%)	IG: (n = 29) Loan of a manual breast pump and educational training session on its use at the hospital PP CG: (n = 30) Educational session on shared reading with the child	EBF at 3 months: IG (n = 16): 31.0% CG (n = 15): 47.0% (*p* = 0.5)	Academic Pediatric Association in Underserved Communities young Investigator Grant ^3^	None declared	++
Panahi et al., 2022 [34]	Randomized controlled trial; Iran (Karaj)	Mean age: Mothers: IG: 21.7 ± 6.7; CG: 22.3 ± 6.6; Fathers: IG: 29.3 ± 5.9; CG: 29.4 ± 8.0 Education level: Mothers: University (Academics) IG + CG mothers: 50.1; Fathers IG: 47.3%; CG: 34.2% Income level: Low as reported by the authors (IG: 76.3%; CG: 81.6%) Intention to breastfeed: NE Marital status: With a partner in majority Ethnicity: NE	IG: (n = 38 couples) Two individual support training sessions with the partner (40 min) during weeks 2–3 PP by the researcher (midwife) CG: (n = 38 couples): Same as IG without the partner	EBF at 4 months: IG: 84.2% CG: 21.1% (*p* < 0.001)	Research deputy of Shahid Beheshti University of Medical Sciences	None declared	−
Shariat et al., 2018 [43]	Randomized controlled trial; Iran (Tehran)	Mean age: IG: 28.6 ± 5.3; CG: 28.5 ± 5.6 Education level: Secondary diploma or less (IG: 79.7%; CG: 78.4%) Income level: NE Intention to breastfeed: Yes Marital status: Majority was married Ethnicity: NE	IG: (n = 64) One breastfeeding educational session (self-efficacy) + 1 education training session (audio set and brochures on breastfeeding and parenting) + Therapy in psychology if needed, all during PP CG *: (n = 65) Standard care * Missing information according to workshop modalities (in-person or remote and individually or in group)	EBF at 6 months: IG: 40.9% CG: 23.5% (*p* = 0.015) Breastfeeding duration until 2 years: IG: 33.8% CG: 7.8% (*p* = 0.001)	Research Deputy of the Tehran University of Medical Sciences	None declared	++
Yin et al., 2021 [36]	Randomized controlled trial; China (Guangzhou)	Mean age: IG: 32 ± 7; CG: 31 ± 7 Education level: NE Income level: NE Intention to breastfeed: Yes Marital status: NE Ethnicity: NE	IG: (n = 206) Standard care (breastfeeding education and text messages) + Baby-led self-attachment breastfeeding support by nurse before hospital discharge PP with partner if needed (missing information about the number of training sessions and whether they are conducted individually or in groups) CG: (n = 203) Standard care (Certified with Baby-Friendly hospital Initiative)	EBF at 6 months: IG: 61.7% CG: 43.8% Mean difference: 17.8%; 95% CI [8.3–27.4%] (*p* < 0.001)	None reported	None declared	+

^1^ IG: Intervention group; CG: Control group; PP: Postpartum period; EBF: Exclusive breastfeeding; NE: not evaluated. ^2^ − Low risk of bias; + Moderate risk of bias (some concerns); ++ High risk of bias. ^3^ Authors also reported funding to the investigators from: the Quality, Safety, and Comparative Effectiveness Research Training in Primary Care (QSCERT-PC) Program, he National Center for Advancing Translational Sciences, National Institutes of Health, Building Interdisciplinary Research Careers in Women’s Health award, the National Institute of Child Health and Human Development (NICHD), Office of Research on Women’s Health, Office of Dietary Supplements, and the National Institute of Aging.

## 6. Interventions During a Combination of Prenatal and Postnatal Periods and Duration of Breastfeeding

Sixteen studies out of the thirty-five examined interventions during a combination of prenatal and postnatal periods (Table 3). Among them, eleven were RCTs, one was a non-randomized controlled trial and four were quasi-experimental studies. Ten showed significant findings about improving EBF duration between 3 and 6 months. Of those, six were remote interventions including online platforms and phones calls, and ten were a combination of interventions.

### 6.1. Remote Interventions with Online Platforms and Phone Calls

A website (e-health) with breastfeeding education content aimed at the mothers and their partner did not show a significant difference in EBF rates at 26 weeks in a study from Abbass-Dick et al. [45]. However, another study from Gonzalez-Darias et al. showed a significant impact on breastfeeding rates at 6 months postpartum among the IG when given access to an educational website, an impact that was even stronger with individualized peer support [46]. Participants from both studies intended to breastfeed and had access to additional support from peers. Support mobile phone applications have also been proposed in two studies; yet, neither of them found a significant impact on EBF duration [47,48]. The first intervention was a comparison of two digital interventions including an interactive educational application (Breastfeeding friend) containing on-demand videos compared to another control application with digital versions of breastfeeding handouts [48]. The second intervention by Bunik et al. compared an educational application offering, from the third trimester until 3 months postpartum, both daily messages and videos or physician-led education via a Facebook group [47]. Cangol at al. proposed a breastfeeding motivation program consisting of four midwife-led phone calls offered between the 32nd and 36th weeks of pregnancy until the 4th month postpartum; yet, they found no significant effect on EBF rates compared to the CG [49]. Puharic et al. also tested an intervention including four support phone calls starting in the prenatal period and lasting up to 2.5 months postpartum. The intervention, which also included printed guides on pregnancy and breastfeeding during the prenatal period, led to significantly higher EBF at 6 months in the IG [50].

**Table 3:** Interventions during a combination of prenatal and postnatal periods to improve breastfeeding duration ^1^.

Authors, Publication Date	Research Design; Study Location	Sociodemographic Characteristics	Sample Size, Interventions	Findings on Breastfeeding Duration	Funding Sources	Conflicts of Interest	Quality Assessment ^2^
Abbass-Dick et al., 2020 [45]	Randomized controlled trial; Canada (Ontario)	Mean age: >31 (IG: 65%; CG: 59%) Education level: Attended university (IG: 74.5%; CG: 74.8%) Income level: >60,000 $, annual income (IG: 54.7%; CG: 55.9%) Intention to breastfeed: Yes Marital status: Married (IG: 82.1%; CG: 78.9%) Ethnicity: Born in Canada (IG: 75.5%; CG: 74.8%)	IG (n = 56 couples): Website e-Health with breastfeeding education content + Available community resources PP CG (n = 56 couples): Available community resources only PP	EBF until 26 weeks: IG: 34.0% CG: 39.0% (*p* = 0.61) Any breastfeeding at 52 weeks: IG (n = 55 couples): 71.0% CG (n = 54 couples): 78.0% (*p* = 0.41)	Women’s Xchange Grant and Women’s College Hospital clinical trial	None declared	−
Bunik et al., 2022 [47]	Randomized controlled trial; United States, (sampling from 44 states)	Mean age: IG: 28.1 ± 5.2; CG: 28.2 ± 5.1 Education level: College (IG: 32%; CG: 29%) Income level: NE Intention to breastfeed: Yes Status marital: Married in majority (IG: 66%; CG: 59%) Ethnicity: White in majority	IG 1: (n = 154) Digital Milk messaging application: Sending daily messages or videos (last 3–4 weeks of pregnancy until 3 months PP) IG 2: (n = 156) Same as IG 1 + Educational content shared via a private Facebook group managed by a physician Combination of the 2 IGs (n = 310) CG: (n = 118) Preventive injury text messages sent on the cellphone	Estimated proportion of EBF at 3 and 6 months (95% CI) (IG1 and IG2 were combined): IG: 58% (49–67%) and 47% (38–56%) CG: 54% (42–66%) and 40% (29–53%) (*p* value for interaction = 0.79)	W.K. Kellogg Foundation	None declared	+
Cangol et al., 2017 [49]	Randomized controlled trial and experimental study; Turkey (Usak)	Mean age: IG: 22.6± 4.5; CG: 22.6 ± 4.3 Education level: ≤8 years in majority Income level: Moderate level, according to authors Intention to breastfeed: Yes Marital status: Married Ethnicity: NE	IG: (n = 34) Four breastfeeding educational and motivational sessions by the researcher (midwife): (1) Between 32 and 36 weeks of pregnancy; (2) Day 1 PP; (3) Between 4 and 6 weeks PP; (4) 4 months PP (by phone) CG: (n = 33) Breast self-examination training session	EBF at 4 months: IG: 61.8% CG: 57.6% (Difference not significant according to the authors)	None reported	None declared	−
Gonzalez-Darias et al., 2020 [46]	Randomized controlled trial; Spain (Canarias)	Mean age: 26–35 years old (IG: 60%; CG: 61%) Education level: Attended university (IG: 51%; CG: 48%) Income level: NE Intention to breastfeed: Yes Marital status: The majority have a life partner Ethnicity: NE	IG: (n = 75) Standard care + Access to a breastfeeding educational website + Individual support with a peer if needed CG: (n = 75) Standard care	EBF at 3 months: IG: 76.0% GC: 56.0% (*p* = 0.020) EBF at 6 months: IG: 60.0% CG: 44.0% (*p* = 0.019)	Canarian Research Foundation	None declared	+
Hans et al., 2018 [51]	Randomized controlled trial, United States (Chicago)	Mean age: IG: 18.3 ± 1.6; CG: 18.5 ± 2.0 Education level: Years of school completed (IG: 10.9; CG: 10.9) Income level: Mostly enrolled in the WIC Program ^3^ (IG: 87.8%; CG: 84.0%) Intention to breastfeed: NE Marital status: Partnered with baby’s father (IG: 68.6%; CG: 72.4%) Ethnicity: African American (45%), Latina (38%), White (8%) and others (9%)	IG: (n = 139) Prenatal classes by doulas + Weekly visits at home by a doula (in majority) during pregnancy, at birth and the firsts weeks PP and by a home visitor, in majority, (Family support worker or parent educator) at 6 weeks until 3 months PP CG: (n = 139) Information was given about case management services in the community	EBF at 3 months: IG: 16.9% CG: 21.8% OR: 0.85, 95% CI [0.45–1.60]	Maternal Infant Early Childhood Home Visiting Competitive grant program from the HRSA, IDHS	None declared	−
Huynh et al., 2018 [52]	Prospective, randomized, open-label, parallel-group, multicenter trial; Vietnam (Haiphong, Hanoï, Ninh Binh et Thai Nguyen)	Mean age: IG: 23.9 ± 2.7; CG: 24.1 ± 3.0 Education level: Secondary and High School (IG: 62%; CG: 60.2%) Income level: NE Intention to breastfeed: NE Marital status: NE Ethnicity: NE	IG: (n = 113) One group breastfeeding support workshop (60 min) during the 3rd trimester of pregnancy + Support visit within 48 h PP + 1 follow-up phone call after 1 week PP + 1 individual follow-up at week PP. All visits conducted by a lactation consultant. Nutritional supplementation offered to mothers CG: (n = 113) Standard care	EBF at 3 months: IG: 41.3% CG: 29.0% OR: 2.09, 95% CI [1.06–4.13] (*p* = 0.04)	Abbott Nutrition	A private company in nutrition	−
Jiang et al., 2014 [53]	Quasi-experimental design; China (Shanghai)	Mean age: 25–29 years old (59.5%) Education level: Diploma ≥ College (86.4%) Income level: (¥) ≥8000 (64.3%) Intention to breastfeed: NE Marital status: NE Ethnicity: NE	IG: (n = 281 from 2 Community health centers) Standard care + Weekly text messages about breastfeeding sent from the 3rd trimester of pregnancy up to 12 months PP CG: (n = 301 from 2 Community health centers) Standard care for 1 year PP	EBF at 6 months: IG (n = 265): 15.1% CG (n = 284): 6.3% RR 2.67, 95%CI [1.45–4.91] Breastfeeding duration at 12 months: IG (258): 20.2% CG (n = 261): 19.2% RR 1.03, 95%CI [0.65–1.63]	Nestle Foundation and Shanghai Municipal Health Bureau’s grant	None declared	+
Ke et al., 2018 [54]	Two-group quasi-experimental design; China (Wuhan)	Mean age: >20 (According to inclusion criteria) Education level: University or above (67.8%) Income level: >5000 ¥ (79.7%) Intention to breastfeed: NE Marital status: Life partner in majority Ethnicity: Han in majority	IG: (n = 29) Two prenatal breastfeeding education sessions including 2 brochures + 3 home visits during the first month PP + 8 support phone calls or text messages every 2 weeks between 2 and 6 months PP The partners and grandmothers were included CG: (n = 30) Standard care + follow-up at 14 days PP by a nurse	EBF at 6 months: IG: 48.3% CG: 16.7% OR = 0.44, 95% CI: [0.20–0.98]	None reported	None declared	+
Lewkowitz et al., 2020 [48]	Randomized controlled trial; United States (Washington)	Mean age: IG: 22.7 ± 4.9; CG: 21.6 ± 4.0 Education level: Secondary (IG: 50%; CG: 45.9%); College (without diploma) (IG: 25%; CG: 30.6%) Income level: <25,000% (IG: 56.0%; GC: 57.7%) Intention to breastfeed: Yes (IG: 51.2%; CG: 57.6%) Marital status: Married or with a partner (IG: 65.5%; CG: 60.2%) Ethnicity: Black (IG: 84.5%; GC: 78.8%), White (IG: 10.7%; CG: 11.8%)	IG: (n = 84) Standard care (Meeting with a lactation consultant at least once + Support group if needed PP) + Breastfeeding Friend (BFF) mobile application including educational and interactive content, on-demand videos about breastfeeding starting during the prenatal period * GC: (n = 85) Standard care + educational breastfeeding mobile application containing brochures usually provided electronically, during the prenatal period (Certified with Baby-Friendly hospital Initiative) * Lack of information on the duration of time mothers used the application PP	EBF at 6 months: IG (n = 60): 8.3% CG (n = 67): 10.4% RR: 0.80, 95% CI [0.27–2.38] (*p* = 0.7)	Washington University in St-Louis’s Institute of Clinical and Translational Sciences Grant	None declared	++
Meedya et al., 2014 [55]	Quasi-experimental design; Australia (Sydney)	Mean age: 26.6 ± 4.9 Education level: University or certificates (IG: 76%; CG: 65.1%) Income level: 50,000 AUD (IG: 52.8%; CG: 49.2%) Intention to breastfeed: Yes (83.6%) Marital status: Married (85.5%) Ethnicity: Born in Australia (IG: 44.2%; CG: 48.7%); Born outside Australia (IG: 48.4%; CG: 41.3%, Non-aboriginal in majority)	IG: (n = 149) Standard care (Follow-ups + 2 group workshops in prenatal period and PP by the obstetric medical team or midwives) + Milky Way program with 3 group workshops with the partner (90 min) during the 2nd trimester of pregnancy + Practical brochures + 2 support phone calls (1) 10 days PP and (2) 3 months PP CG: (n = 189) Standard care	EBF at 6 months: IG (n = 94): 19.1% CG (n = 61): 6.6% (*p* = 0.045)	None reported	None declared	+
Prasitwattanaseree et al., 2019 [56]	Randomized controlled trial; Thailand (Northern Thailand)	Mean age: IG: 27.9 ± 4.6; CG: 27.1 ± 4.7 Education level: Attended university (IG: 58.5%; GC: 50%) Income level: IG: 26,024 ± 10,607 ฿; CG: 22,269 ± 8230 ฿ Intention to breastfeed: Yes Marital status: Married Ethnicity: NE	IG: (n = 41) Standard care (1 educational training session during the prenatal period + 1 group workshop PP) + Breastfeeding education training sessions (45 min, 1 x/week for 2 weeks) from 36 to 37 weeks of pregnancy + Breastfeeding support at hospital by the researcher (nurse) with the partner if needed (Days 1, 2 and 3 and at week 6 PP) + 5 Support phone calls (10–20 min) (Day 7, at 1 month and 1 x/month from 3 to 6 months PP) CG: (n = 42): Standard care	EBF at 6 months: IG: 36.6% CG: 14.3% (*p* = 0.01)	None reported	None declared	−
Puharic et al., 2020 [50]	Single-centre, randomized controlled, three-arm, superiority study; Croatia (Split)	Mean age: 25–35 years old (IG: 71%; CGA: 70%; CG: 59%) Education level: Secondary (IG: 47%; CGA: 41%; CG: 45%), University (IG: 36%; CGA 39%; CG: 41%) Income level: 472–950 €/months (IG: 74%; CGA 68%: CG: 68%) Intention to breastfeed: Yes Marital status: With a partner Ethnicity: NE	IG: (n = 129) Breastfeeding guide + Pregnancy guide given during prenatal period + 4 follow-up phone calls by a nurse (1 prenatal and at weeks 2, 6 and 10 PP) Control group with attention (CGA): (n = 103) Pregnancy guide + 4 follow-up phone calls as IG CG: (n = 123) Standard care (No guide or phone calls)	EBF at 6 months (Imputed data): IG: 60.0% CGA: 32.0% CG: 17.0% (IG RR: 18.3, 95% CI [10.7–31.2]; CGA RR: 1.8, 95% CI [1.1–2.9]; CG: Reference)	None reported	None declared	−
Rosuzeita et al., 2018 [57]	Quasi-experimental design; Malaysia (Kelantan)	Mean age: 25.7 ± 4.1 Education level: Secondary school (51%), higher education (46.9%) Income level: 2696 ± 1799 MYR Intention to breastfeed: Yes Marital status: NE Ethnicity: Malay in majority	IG: (n = 48) Standard care + 1 breastfeeding workshop (breastfeeding guide and videos) 28th week of pregnancy + Breastfeeding support 1st week PP at the hospital by the researcher (nurse) CG: (n = 46) Standard care (Certified with Baby-Friendly hospital Initiative)	EBF at 4 months: IG: 54.3% CG: 29.5% (*p* = 0.02) EBF at 6 months: IG (n = 44): 27.3% CG (n = 42): 16.7% (*p* = 0.24)	University Teknologi MARA and Ministry of Higher Education	None declared	+
Yi et al., 2016 [58]	Longitudinal randomized controlled trial; China (Hong-Kong)	Mean age: IG: 32.6 ± 3.5; CG: 31.4 ± 4.2 Education level: Bachelor’s degree (IG: 45.7%; CG: 44.4%) Income level: (HK$) 15,000–25,000 (IG: 41.7%; GC: 45.7%) Intention to breastfeed: Yes Marital status: Married Ethnicity: Chinese mothers (95%)	IG: (n = 35) Standard care (Breastfeeding support at hospital by midwives, lactation consultant if needed + follow-ups by physician or midwives PP) + 1 breastfeeding workshop in group (90 min) between 28 and 38 weeks of pregnancy + 1 counseling phone call (30–60 min) at week 2 PP GC: (n = 36) Standard care	EBF at 6 months (included breast milk expression): IG: 11.4% CG: 5.6% (*p* = 0.34)	Association of Hong Kong Nursing Staff Grant	None declared	−
Yilmaz et al., 2021 [59]	Randomized controlled trial; Turkey (Kayseri)	Mean age: IG: 23 CG: 24.5 Education level: Secondary school and high school (IG: 67.7%; CG: 66.7%) Income level: Between 1400 and 1500 ₺ Intention to breastfeed: NE Marital status: With a partner in majority Ethnicity: NE	IG: (n = 34) One prenatal group breastfeeding workshop (90 min) + 1 education training session at hospital with the partner or family (30 min, day 1 PP) + Home visit by the researcher (Department of nutrition) (Week 1 PP) CG: (n = 30) Standard care (Certified with Baby-Friendly hospital Initiative)	EBF at 6 months: IG: 26.5% CG: 3.3% (*p* < 0.02)	Erciyes University Coordination of Scientific Research Projects	None declared	−
Zhang et al., 2021 [60]	Non-randomized controlled trial; China (Nantong)	Mean age: IG: 26.9± 4.5; CG: 26.6 ± 4.4 Education level: Secondary school or less (IG: 37.1%; CG: 38.6%), College or university (IG: 31.4%; CG: 32.9%) Income level: >4000 ¥ (IG: 95.7%; CG: 95.7%) Intention to breastfeed: NE Marital status: NE Ethnicity: NE	IG: (n = 70) One group prenatal breastfeeding educational session (based on the Theory of planned behavior) (30 min) + Weekly access to an online support group (WeChat) + Support session (15 min, 2 x/day) at hospital PP + Support phone calls (at hospital discharge and at 4 months) PP + Breastfeeding discussion groups in-person at 4 months, all provided by medical staff, with the partner or family CG: (n = 70) Standard care	EBF at 4 months: IG: 58.6% CG: 41.4% (*p* = 0.04)	None reported	None declared	++

^1^ IG: Intervention group; CG: Control group; CGA: Control group with attention; PP: Postpartum period; EBF: Exclusive breastfeeding; NE: not evaluated; HRSA: Health Resources and Services Administration; IDHS: Illinois Department of Human Services. ^2^ − Low risk of bias; + Moderate risk of bias (some concerns); ++ High risk of bias. ^3^ Women, Infants, and Children (WIC) Program is for low-income women, infants, and children up to age 5 who are at nutrition risk.

### 6.2. Combination of Interventions

Ke et al. examined the effect of an intervention consisting of two breastfeeding education group sessions, a guide during the prenatal period, home visits during the first month postpartum with partners or grandmothers and the possibility of support phone calls or text messages until 6 months postpartum [54]. Significantly more women were exclusively breastfeeding their child at 6 months in the IG compared to the CG [54]. A similar intervention offered two breastfeeding education group sessions during pregnancy, follow-ups in the hospital for the parents with a nurse during the first 2 months in the postpartum period and access to support phone calls until 6 months postpartum and also found a significantly longer duration of EBF in the IG at 6 months compared to the CG [56]. Significant and positive effects on EBF rates were also found in two studies testing similar combinations of interventions including group-support workshops [52] or in-hospital support visits after delivery and virtual support group with WeChat [60].

Two studies examined the effect of one breastfeeding group workshop with the distribution of a guide during the prenatal period [57,59]. Rosuzeita et al. also offered an in-hospital support follow-up led by a nurse for mothers within their first week postpartum, and EBF rates were significantly higher in the IG compared to the CG at four months postpartum [57]. The other study from Yilmaz et al. proposed an intervention with two follow-up visits for mothers and their partner within their first week postpartum and found significantly higher EBF rates at 6 months in the IG compared to the CG [59]. Meedya et al. compared a CG to an IG with three workshops offered to mothers and their partner, a booklet and phone calls for the first three months postpartum, and they found significantly higher EBF rates at 6 months in the IG compared to the CG [55]. No significant effect at 6 months postpartum was found by Yi et al. for an intervention combining one prenatal breastfeeding group workshop with one support phone call at two weeks postpartum [58]. Finally, Hans et al. is the only study that proposed prenatal classes with doulas and weekly in-person visits at home (not by phone) from pregnancy up to three months postpartum. In this low-income community, EBF rates of the IG at three months were low and non-significantly different compared to the CG [51].

## 7. Sociodemographic Factors and the Efficacy of Interventions

Six studies have explored the role of sociodemographic characteristics in breastfeeding duration in the context of interventions. In a study from Gonzalez-Darias et al. conducted in Spain, mothers were given access to an educational website and support with a peer, and only two characteristics were statistically associated with breastfeeding rates [46]. Older maternal age (33 ± 5 vs. 30 ± 5 years old) was significantly associated with higher breastfeeding rates at 3 months, while higher maternal education level (university vs. compulsory education) was significantly associated with higher breastfeeding rates at 3 and 6 months [46]. Hermanson et al. analyzed the probability of ceasing to breastfeed before 6 months after birth when offering a pacifier from the first day of life compared to avoiding it during the first two weeks of life. Three maternal characteristics were positively significantly associated with a higher proportion of breastfeeding cessation before 6 months: intention to breastfeed under 6 months, no university education and use of nipple shields for the whole lactation duration compared to no use [38]. Meedya et al. also adjusted for some predicting factors of prolonged breastfeeding and found increased breastfeeding rates at 6 months in older women and in women with intention to breastfeed for 6 months [55]. Abbott et al. made similar adjustments for confounding variables at 5–6 months postpartum, and only women who planned EBF and who had a vaginal delivery breastfed for a significantly longer duration [35]. Puharic et al. found significantly increased EBF rates at 3 months in women older than 25 years old and in women with no intention to use a pacifier [50]. Surprisingly, a negative association was found at 6 months for EBF rates and women’s intention to breastfeed up to 4–6 months [50]. Ke at al. proposed a combination of interventions (i.e., reading materials, home visits and phone calls with the partners or grandmothers) and examined predictive factors of EBF. Only early maternal–infant separation showed a significant association with lower EBF duration [54]. Few studies examined sociodemographic characteristics associated with breastfeeding duration in their interventions, but overall, older maternal age, higher education level and intention to breastfeed showed positive associations with longer breastfeeding rates [35,38,46,50,55].

## 8. Discussion

This scoping review examined the effectiveness of different types of interventions to improve breastfeeding duration among primiparous women. The interventions were heterogeneous and offered during the prenatal and the postnatal periods, or in a combination of both periods. Intervention, delivery method, frequency of visits during the different periods of intervention and intervention providers varied greatly between studies. No two interventions were the same; however, the similarities of certain interventions made the comparisons between them possible. Most studies examined the impact on EBF at 3 or 6 months, and only some studies evaluated the impact on breastfeeding between 12 and 24 months [43,45,53].

In the prenatal period, studies showed benefits on EBF duration at 4 and 6 months with group workshops delivered solely in the prenatal period. Between one and four workshops were proposed in the third trimester led by breastfeeding educators, nurses, midwives or peers, and EBF rates were between 32 and 73% compared to control groups (13–27%) [26,27,28,29]. A study from Oberfichtner et al. showed that stronger maternal desire to breastfeed and sufficient knowledge about breastfeeding before childbirth were associated with a longer duration of breastfeeding. The pressure felt by primiparous mothers can be greater, and the support they receive afterward can greatly influence the duration of breastfeeding, highlighting the importance of combining prenatal and postnatal interventions for this population [61].

Many interventions were also combined with a breastfeeding booklet. Among them, six studies showed a positive and significant impact on EBF duration until 4 to 6 months (rates between 27 and 82%, compared to control groups 6–57%) [26,28,31,50,54,57]. It should be noted that the quality of the studies and the combination of interventions were variable; therefore, the contribution of the breastfeeding booklet alone on EBF duration could not be determined, but it may help in combination with interventions such as workshops during the prenatal period. Lumbiganon et al. conducted a review of systematic reviews on antenatal breastfeeding education and, similarly, found that the following interventions marginally improved EBF rates at 6 months: education sessions (RR: 1.02, 95%CI [0.8–1.3]), education workshops (RR: 1.1, 95%CI [0.7–1.8]) and combination of breastfeeding booklet, video and lactation consultants (OR: 2.4, 95% CI [1.0–5.8]) [62]. Fewer studies in our review have analyzed educational websites or a mobile application, and only two showed a significant impact with breastfeeding rates around 60–82% at 6 months [31,46] and another one with a rate of 71% after one year (nonsignificant) [44,45,47,48]. Breastfeeding applications and text messages showed the lowest benefit on EBF rates (between 8% and 47%) even when considering the mothers’ frequency of use or intention to breastfeed [47,48,53,63,64]. On the other hand, Hauck et al. showed that digital support is perceived differently in terms of its importance for continuing breastfeeding across countries (Australia, Irish and Swedish and with a combination of parity), highlighting the need to combine different interventions according to parents’ needs [65].

Our review also demonstrated that remote support, i.e., motivational or educational interventions mostly by phone calls by peers or professionals, could also improve breastfeeding duration. Most of them were used in combination with other intervention(s), but only two looked at motivational support phone calls during the postpartum period, and two others in combination with the third trimester and postpartum period [39,40,49,50]. Intensive phone calls were conducted mostly in the first 3 months postpartum, and on average twice during the first month and once thereafter [39,40,41,42,49,50,52,54,55,56,60]. These results are similar to the systematic review by Corkery-Hayward et al. which indicated that teleinterventions (peer support or educational phone calls) in five RCTs modestly increased EBF rates at 6 months (RR: 1.3, 95% CI [0.8–2.1]) [66]. However, the included studies were conducted only in the United States with small sample sizes of low-income women and had high risk of bias. Another systematic review and meta-analysis from Sun et al. showed similar findings with twelve RCTs with significantly higher EBF rates at 6 months with remote interventions (phone calls, text messages), on average from the last trimester of pregnancy to 6 months in the postnatal period (RR: 1.7, 95% CI [1.5–2.02]) [67]. The studies included in this systematic review by Sun et al. were comparable with the ones included in our scoping review in terms of countries of studies and sample sizes, but there was a significant heterogeneity among the results according to the authors. Also, these previous reviews included both primiparous and multiparous women, which limits the comparison with our review.

Individual educational or support interventions by professionals (nurses, IBCLCs, midwives, doulas or physicians) during both the prenatal and postnatal periods were also beneficial. Three studies, between 4 and 6 months, showed significant results on EBF rates around 50–84% [33,34,36] and others with low–moderate rates around 17–78% between 3 and 6 months [30,35,51]. Most studies proposed interventions between the third trimester of pregnancy and three weeks in the postpartum period [30,33,34,35,36]. We only found one systematic review from Wong et al. comparing effectiveness between individual and group education in the prenatal period [68]. No conclusive associations were found, mostly due to heterogeneity between the studies and low study quality [68]. Two of the eighteen studies analyzed were part of our review, and most of them included multiparous mothers, which highlights the needs for doing a scoping review with primiparous women only. Yet, group or individual education support during the prenatal period from a professional, combined with individual support during the first postpartum months, seems to be a promising solution.

A combination of interventions was also tested in multiple studies during the postnatal period or as a combination of prenatal and postnatal periods. In most studies included in our scoping review, there was a combination of education and support. There were group workshops during the prenatal period and/or in the first week after delivery with, on average, two individual support follow-ups in the first week at the hospital from a professional (nurses, breastfeeding consultants, physicians). Support phone calls and/or individual follow-ups at home within the first month were also provided to mothers and sometimes partners, and then, on average, less intensively for up to 3–6 months [41,52,54,55,56,57,58,59,60]. Seven studies showed significant effects on EBF rates between 3 and 6 months (around 19–59%) [41,52,54,55,56,59,60] with two others with low EBF rates at 6 months (around 11–27%) [58]. Yet, some studies had small sample sizes, moderate quality, and, although significant, the EBF rates observed were not that high [41,52,54,55,56,57,59,60]. Skouteris et al. conducted a systematic review including interventions to improve breastfeeding duration and found that a combination of support-based interventions (home visits, phone calls) during the postnatal period from birth to 6 months showed greater benefits compared to one education intervention alone, short-term interventions or at-hospital before discharge interventions [69]. These findings are somewhat similar to our findings; however, studies in this systematic review included multiparous and primiparous women from high-income countries, which limits comparison.

The partner or family’s participation had a positive impact on improving EBF duration in the studies that combined interventions during both the prenatal and postnatal periods (EBF rates between 19 and 43%) [54,55,56,59,60]. Four studies also included partners during postnatal interventions and showed a significant impact at 4–6 months (42–84%); only Abbass-Dick et al. had nonsignificant findings for EBF rates at 3 months (IG: 67.4%) [34,36,41,44]. Similarly, three other studies showed significant effects on EBF rates at 6 months with only prenatal interventions (35–50%) [28,29,33]. This is consistent with a recent systematic review and meta-analysis by Zhao et al. (n = 8 articles) that showed significant benefits of including partners in the interventions on EBF rates at 6 months (OR = 2.8, 95% CI [1.5 to 5.4], *p* = 0.002, I^2^ = 85%) [70]. Grandmothers can also positively or negatively influence breastfeeding practices according to their perception and attitude towards breastfeeding as specified in the systematic review by Negin et al. [71]. Thus, including the participation of partners or family members in the intervention could positively influence long-term breastfeeding outcomes.

Only a few studies have analyzed the impact of sociodemographic characteristics on breastfeeding duration in the context of interventions. These studies showed that older maternal age, higher education level and intention to breastfeed were positively associated with EBF duration during these interventions [35,38,46,50,55]. A systematic review from Mangrio et al. revealed significant associations between shorter breastfeeding duration and younger maternal age, low maternal education and mothers returning to work early [11]. Even if this systematic review is from 2013, with mostly observational design studies, results aligned with those observed in the studies included in our scoping review. Nine studies in our review included content about breastfeeding and returning to work in their interventions (text messages, education content, support, etc.) [27,32,41,46,47,48,53,54,55]. However, the effectiveness of the interventions is difficult to assess, but it appears to emphasize the importance of providing long-term support. Most of them were from the United States and China. Also, no included study has explored the impact of ethnicity and income on EBF rates. However, one study examined how ethnicity and cultural sensitivity should be assessed within a breastfeeding intervention targeting black immigrant mothers. The authors suggested considering determinants at multiple levels, including individual (“knowledge and perception of breastfeeding”), interpersonal (“influence of peers”), organizational (“workplace”) and sociocultural (“stigma of breastfeeding in public or workplace”) determinants. They also emphasized the importance of being aware of our cultural bias or prejudices when an intervention is offered by fostering a dialogue to better understand parents’ perceptions, attitude and challenges related to breastfeeding [72,73]. A descriptive phenomenological study conducted by Nan et al. also showed that interventions by professionals must be tailored on mothers’ needs. It is necessary to recognize that sometimes they may not reach for professional help according to personal, feasibility or cultural reasons [74]. These dimensions should be further analyzed to make each intervention more tailored.

This scoping review has many strengths. First, it is the first review on this topic to focus only on primiparous women. Also, the risk of bias has been assessed for each study to enhance the validity of the results’ interpretation [22]. Most studies included in the scoping review are RCTs, and many included women that had the intention to breastfeed (n = 17). On the other hand, this may have biased the studies’ results by increasing the desirability in participating in research, increasing the effectiveness of the interventions. The scoping review includes studies from several countries, which gives an overview of all the interventions that may yet exist. However, there was some heterogeneity in standard of care, with only 12 out of the 35 studies that described the standard care provided to mothers. This scoping review also has some limitations. There was an important diversity of interventions, which limited comparisons. Additionally, many studies were assessed as having a high risk of bias, which may restrict the generalizability of the findings. Few studies have examined the impact of sociodemographic characteristics on breastfeeding duration, highlighting the need for further research on the intervention’s effectiveness for primiparous women in various sociodemographic contexts.

The most effective approach to increase breastfeeding duration among primiparous women remains uncertain. A variety of interventions, delivered in both the prenatal and postnatal periods, and including partners, seems to be the most promising approach to increase the duration of breastfeeding. A combination of group workshops or individual education with support sessions during the last trimester of pregnancy tends to have a positive impact on EBF [26,28,29,30,33]. The addition of support interventions is also favorable for EBF with professionals or peers (phone calls, text messages, home visits). This had a positive impact on breastfeeding duration when implemented more intensively during the first few months (weekly) and then decreased gradually in frequency, while remaining available until at least 6 months [34,36,39,40,41,42,49,50,53,54,56,57,60]. Also, it seems relevant to focus on the mothers’ intentions to breastfeed during the prenatal period and, as much as possible, to target younger primiparous mothers with lower levels of education with our interventions [35,38,46,50,55].

## 9. Conclusions

To summarize, a combination of support and educational interventions by professionals or peers during the prenatal and postnatal periods seem to be more effective to improve EBF rates among primiparous mothers. Primiparous young women with low education levels should be targeted, and inclusion of their partner or a family member during the intervention is also to be considered. Future research could further examine the role of sociodemographic characteristics (ethnicity, culture, etc.) in the effectiveness of breastfeeding interventions to better tailor interventions in different contexts. Additionally, more studies should be conducted with interventions that extend over a longer period, beyond 6 months, as the challenge to maintain breastfeeding persists over one year. Our scoping review was a first step in analyzing the current state of knowledge, but it remains difficult to conclude about the most effective way to deliver interventions in terms of frequency and modalities due to heterogeneous outcomes.

## Data Availability

All data generated or analyzed during this study are included in this published article and are all listed in the references.

## Supplementary Materials

The following supporting information can be downloaded at: https://www.mdpi.com/article/10.3390/pediatric18020035/s1, Table S1: Final MEDLINE Search Strategy; Table S2: Risk of bias domains: author judgments about each RCT included in the scoping review; Table S3: Risk of bias: author judgments about non-randomized studies of interventions included in the scoping review. Preferred Reporting Items for Systematic reviews and Meta-Analyses extension for Scoping Reviews (PRISMA-ScR) Checklist.

## Abbreviations

The following abbreviations are used in this manuscript:

CI	Confidence interval
CG	Control group
EBF	Exclusive breastfeeding
IG	Intervention group
RCT	Randomized controlled trial
RR	Risk ratio
UNICEF	United Nations International Children’s Emergency Fund
WHO	World Health Organization

## References

[1] World Health Organization (2024). Breastfeeding Recommendations.

[2] World Health Organization (2023). Exclusive Breastfeeding for Optimal Growth, Development and Health of Infants.

[3] Ahsan S., Jain S., Walters D. (2022). The Global Cost of Not Breastfeeding.

[4] Sankar M.J., Sinha B., Chowdhury R., Bhandari N., Taneja S., Martines J., Bahl R. (2015). Optimal breastfeeding practices and infant and child mortality: A systematic review and meta-analysis. Acta Paediatr..

[5] Victora C.G., Bahl R., Barros A.J., França G.V., Horton S., Krasevec J., Murch S., Sankar M.J., Walker N., Rollins N.C. (2016). Breastfeeding in the 21st century: Epidemiology, mechanisms, and lifelong effect. Lancet.

[6] Horta B.L., de Lima N.P. (2019). Breastfeeding and Type 2 Diabetes: Systematic Review and Meta-Analysis. Curr. Diabetes Rep..

[7] Horta B.L., Rollins N., Dias M.S., Garcez V., Pérez-Escamilla R. (2023). Systematic review and meta-analysis of breastfeeding and later overweight or obesity expands on previous study for World Health Organization. Acta Paediatr..

[8] Chowdhury R., Sinha B., Sankar M.J., Taneja S., Bhandari N., Rollins N., Bahl R., Martines J. (2015). Breastfeeding and maternal health outcomes: A systematic review and meta-analysis. Acta Paediatr..

[9] Global Breastfeed Collective, UNICEF and WHO (2023). Rates of Breastfeeding Increase Around the World Through Improved Protection and Support.

[10] Cohen S.S., Alexander D.D., Krebs N.F., Young B.E., Cabana M.D., Erdmann P., Hays N.P., Bezold C.P., Levin-Sparenberg E., Turini M. (2018). Factors Associated with Breastfeeding Initiation and Continuation: A Meta-Analysis. J. Pediatr..

[11] Mangrio E., Persson K., Bramhagen A.-C. (2018). Sociodemographic, physical, mental and social factors in the cessation of breastfeeding before 6 months: A systematic review. Scand. J. Caring Sci..

[12] Wagner S., Kersuzan C., Gojard S., Tichit C., Nicklaus S., Thierry X., Charles M.A., Lioret S., de Lauzon-Guillain B. (2019). Breastfeeding initiation and duration in France: The importance of intergenerational and previous maternal breastfeeding experiences—Results from the nationwide ELFE study. Midwifery.

[13] Huang Y., Ouyang Y.Q., Redding S.R. (2019). Previous breastfeeding experience and its influence on breastfeeding outcomes in subsequent births: A systematic review. Women Birth.

[14] Schafer E.J., Campo S., Colaizy T.T., Mulder P.J., Ashida S. (2017). Influence of Experiences and Perceptions Related to Breastfeeding One’s First Child on Breastfeeding Initiation of Second Child. Matern. Child Health J..

[15] Buckland C., Hector D., Kolt G.S., Fahey P., Arora A. (2020). Interventions to promote exclusive breastfeeding among young mothers: A systematic review and meta-analysis. Int. Breastfeed. J..

[16] Patel S., Patel S. (2016). The Effectiveness of Lactation Consultants and Lactation Counselors on Breastfeeding Outcomes. J. Hum. Lact..

[17] Pezley L., Cares K., Duffecy J., Koenig M.D., Maki P., Odoms-Young A., Clark Withington M.H., Lima Oliveira M., Loiacono B., Prough J. (2022). Efficacy of behavioral interventions to improve maternal mental health and breastfeeding outcomes: A systematic review. Int. Breastfeed. J..

[18] Sinha B., Chowdhury R., Sankar M.J., Martines J., Taneja S., Mazumder S., Rollins N., Bahl R., Bhandari N. (2015). Interventions to improve breastfeeding outcomes: A systematic review and meta-analysis. Acta Paediatr..

[19] Kusvitasari H., Ismarwati I., Mahmudah N. (2022). Factors affecting exclusive breastfeeding of primiparous mothers: Scoping review. Int. J. Health Sci. Technol..

[20] Whittaker X., Meedya S., Capper T. (2025). Factors and interventions that positively influence breastfeeding rates at six months postpartum: An integrative literature review. Women Birth.

[21] Peters M.D.J., Marnie C., Tricco A.C., Pollock D., Munn Z., Alexander L., McInerney P., Godfrey C.M., Khalil H. (2021). Updated methodological guidance for the conduct of scoping reviews. JBI Evid. Implement..

[22] Tricco A.C., Lillie E., Zarin W., O’Brien K.K., Colquhoun H., Levac D., Moher D., Peters M.D.J., Horsley T., Weeks L. (2018). PRISMA Extension for Scoping Reviews (PRISMA-ScR): Checklist and Explanation. Ann. Intern. Med..

[23] Higgings J. (2019). Revised Cochrane Risk-of-Bias Tool for Randomized Trials (RoB2), Current Version of RoB2. Cochrane Methods Bias. https://www.riskofbias.info/welcome/rob-2-0-tool/current-version-of-rob-2.

[24] Sterne J.A. (2016). The Risk of Bias in Non-Randomized Studies—Of Interventions (ROBINS-1)—Current Version of ROBINS-1. Cochrane Methods Bias. https://methods.cochrane.org/bias/risk-bias-non-randomized-studies-interventions.

[25] The World Bank Group (2024). The World by Income and Region. The World Bank (IBRD-IDA). https://datatopics.worldbank.org/world-development-indicators/the-world-by-income-and-region.html.

[26] Ansari S., Abedi P., Hasanpoor S., Bani S. (2014). The Effect of Interventional Program on Breastfeeding Self-Efficacy and Duration of Exclusive Breastfeeding in Pregnant Women in Ahvaz, Iran. Int. Sch. Res. Not..

[27] Naroee H., Rakhshkhorshid M., Shakiba M., Navidian A. (2020). The Effect of Motivational Interviewing on Self-Efficacy and Continuation of Exclusive Breastfeeding Rates: A Quasi-Experimental Study. Breastfeed. Med..

[28] Su M., Ouyang Y.Q. (2016). Father’s Role in Breastfeeding Promotion: Lessons from a Quasi-Experimental Trial in China. Breastfeed. Med..

[29] Tseng J.F., Chen S.R., Au H.K., Chipojola R., Lee G.T., Lee P.H., Shyu M.L., Kuo S.Y. (2020). Effectiveness of an integrated breastfeeding education program to improve self-efficacy and exclusive breastfeeding rate: A single-blind, randomised controlled study. Int. J. Nurs. Stud..

[30] Demirci J.R., Glasser M., Himes K.P., Sereika S.M. (2022). Structured antenatal milk expression education for nulliparous pregnant people: Results of a pilot, randomized controlled trial in the United States. Int. Breastfeed. J..

[31] Taheri Z., Bakouei F., Delavar M.A., Faramarzi M., Bakhtiari A., Amiri F.N. (2022). Effectiveness of distance education program on mothers’ empowerment in exclusive breastfeeding: A randomized clinical trial. J. Educ. Health Promot..

[32] Wong K.L., Tak Fong D.Y., Yin Lee I.L., Chu S., Tarrant M. (2014). Antenatal education to increase exclusive breastfeeding: A randomized controlled trial. Obs. Gynecol..

[33] Zhao Y., Lin Q., Zhu X., Wang J. (2021). Randomized Clinical Trial of a Prenatal Breastfeeding and Mental Health Mixed Management Intervention. J. Hum. Lact..

[34] Panahi F., Rashidi Fakari F., Nazarpour S., Lotfi R., Rahimizadeh M., Nasiri M., Simbar M. (2022). Educating fathers to improve exclusive breastfeeding practices: A randomized controlled trial. BMC Health Serv. Res..

[35] Abbott J.L., Carty J.R., Hemman E., Batig A.L. (2019). Effect of Follow-Up Intervals on Breastfeeding Rates 5-6 Months Postpartum: A Randomized Controlled Trial. Breastfeed. Med..

[36] Yin C., Su X., Liang Q., Ngai F.W. (2021). Effect of Baby-Led Self-Attachment Breastfeeding Technique in the Postpartum Period on Breastfeeding Rates: A Randomized Study. Breastfeed. Med..

[37] Hoyt-Austin A.E., Cheng J.H., Moua H., Tancredi D.J., Chantry C.J., Kair L.R. (2023). Providing Low-Income Women With a Manual Pump: A Pilot Study. Hosp. Pediatr..

[38] Hermanson A., Astrand L.L. (2020). The effects of early pacifier use on breastfeeding: A randomised controlled trial. Women Birth.

[39] Chehreh R., Zahrani S.T., Karamelahi Z., Baghban A.A. (2021). Effect of peer support on breastfeeding self-efficacy in ilamian primiparous women: A single-blind randomized clinical trial. J. Fam. Med. Prim. Care.

[40] Forster D.A., McLardie-Hore F.E., McLachlan H.L., Davey M.A., Grimes H.A., Dennis C.L., Mortensen K., Moorhead A.M., Tawia S., Gold L. (2019). Proactive Peer (Mother-to-Mother) Breastfeeding Support by Telephone (Ringing up About Breastfeeding Early [RUBY]): A Multicentre, Unblinded, Randomised Controlled Trial. eClinicalMedicine.

[41] Gu Y., Zhu Y., Zhang Z., Wan H. (2016). Effectiveness of a theory-based breastfeeding promotion intervention on exclusive breastfeeding in China: A randomised controlled trial. Midwifery.

[42] Chegeni M.F., Valizadeh F., Ghasemi S.F., Changaee F., Anbari K. (2022). Comparison of Different Virtual Follow-ups on Mother’s Lactation. J. Nurse Pract..

[43] Shariat M., Abedinia N., Noorbala A.A., Zebardast J., Moradi S., Shahmohammadian N., Karimi A., Abbasi M. (2018). Breastfeeding Self-Efficacy as a Predictor of Exclusive Breastfeeding: A Clinical Trial. Iran. J. Neonatol..

[44] Abbass-Dick J., Stern S.B., Nelson L.E., Watson W., Dennis C.L. (2015). Coparenting breastfeeding support and exclusive breastfeeding: A randomized controlled trial. Pediatrics.

[45] Abbass-Dick J., Sun W., Newport A., Xie F., Godfrey D., Goodman W.M. (2020). The comparison of access to an eHealth resource to current practice on mother and co-parent teamwork and breastfeeding rates: A randomized controlled trial. Midwifery.

[46] Gonzalez-Darias A., Diaz-Gomez N.M., Rodriguez-Martin S., Hernandez-Perez C., Aguirre-Jaime A. (2020). ‘Supporting a first-time mother’: Assessment of success of a breastfeeding promotion programme. Midwifery.

[47] Bunik M., Jimenez-Zambrano A., Solano M., Beaty B.L., Juarez-Colunga E., Zhang X., Moore S.L., Bull S., Leiferman J.A. (2022). Mother’s Milk Messaging: Trial evaluation of app and texting for breastfeeding support. BMC Pregnancy Childbirth.

[48] Lewkowitz A.K., Lopez J.D., Carter E.B., Duckham H., Strickland T., Macones G.A., Cahill A.G. (2020). Impact of a novel smartphone application on low-income, first-time mothers’ breastfeeding rates: A randomized controlled trial. Am. J. Obs. Gynecol. MFM.

[49] Cangol E., Sahin N.H. (2017). The Effect of a Breastfeeding Motivation Program Maintained During Pregnancy on Supporting Breastfeeding: A Randomized Controlled Trial. Breastfeed. Med..

[50] Puharic D., Malicki M., Borovac J.A., Sparac V., Poljak B., Aracic N., Marinovic N., Luetic N., Zakarija-Grkovic I. (2020). The effect of a combined intervention on exclusive breastfeeding in primiparas: A randomised controlled trial. Matern. Child. Nutr..

[51] Hans S.L., Edwards R.C., Zhang Y. (2018). Randomized Controlled Trial of Doula-Home-Visiting Services: Impact on Maternal and Infant Health. Matern. Child. Health J..

[52] Huynh D.T.T., Tran N.T., Nguyen L.T., Berde Y., Low Y.L. (2018). Impact of maternal nutritional supplementation in conjunction with a breastfeeding support program on breastfeeding performance, birth, and growth outcomes in a Vietnamese population. J. Matern. Fetal Neonatal Med..

[53] Jiang H., Li M., Wen L.M., Hu Q., Yang D., He G., Baur L.A., Dibley M.J., Qian X. (2014). Effect of short message service on infant feeding practice: Findings from a community-based study in Shanghai, China. JAMA Pediatr..

[54] Ke J., Ouyang Y.Q., Redding S.R. (2018). Family-Centered Breastfeeding Education to Promote Primiparas’ Exclusive Breastfeeding in China. J. Hum. Lact..

[55] Meedya S., Fahy K., Yoxall J., Parratt J. (2014). Increasing breastfeeding rates to six months among nulliparous women: A quasi-experimental study. Midwifery.

[56] Prasitwattanaseree P., Sinsuksai N., Prasopkittikun T., Viwatwongkasem C. (2019). Effectiveness of Breastfeeding Skills Training and Support Program among First Time Mothers: A Randomized Control Trial. Pac. Rim Int. J. Nurs. Res..

[57] Rosuzeita F., Che Rabiaah M., Rohani I., Mohd Shukri O. (2018). The Effectiveness of Breastfeeding Intervention on Breastfeeding Exclusivity and Duration among Primiparous Mothers in Hospital Universiti Sains Malaysia. Malays. J. Med. Sci..

[58] Yi C.H., Yim I.W., Chow C.K. (2016). The effect of a self-efficacy-based educational programme on maternal breast feeding self-efficacy, breast feeding duration and exclusive breast feeding rates: A longitudinal study. Midwifery.

[59] Yilmaz M., Aykut M. (2021). The effect of breastfeeding training on exclusive breastfeeding: A randomized controlled trial. J. Matern. Fetal Neonatal Med..

[60] Zhang Y., Yuan R., Ma H. (2021). Effect of the theory of planned behavior on primipara breastfeeding. Ann. Palliat. Med..

[61] Oberfichtner K., Oppelt P., Fritz D., Hrauda K., Fritz C., Schildberger B., Lastinger J., Stelzl P., Enengl S. (2023). Breastfeeding in primiparous women—Expectations and reality: A prospective questionnaire survey. BMC Pregnancy Childbirth.

[62] Lumbiganon P., Martis R., Laopaiboon M., Festin M.R., Ho J.J., Hakimi M. (2016). Antenatal breastfeeding education for increasing breastfeeding duration. Cochrane Database Syst. Rev..

[63] Griffin L.B., Lopez J.D., Ranney M.L., Macones G.A., Cahill A.G., Lewkowitz A.K. (2021). Effect of Novel Breastfeeding Smartphone Applications on Breastfeeding Rates. Breastfeed. Med..

[64] Lewkowitz A.K., Lopez J.D., Werner E.F., Ranney M.L., Macones G.A., Rouse D.J., Savitz D.A., Cahill A.G. (2021). Effect of a Novel Smartphone Application on Breastfeeding Rates Among Low-Income, First-Time Mothers Intending to Exclusively Breastfeed: Secondary Analysis of a Randomized Controlled Trial. Breastfeed. Med..

[65] Hauck Y.L., Blixt I., Hildingsson I., Gallagher L., Rubertsson C., Thomson B., Lewis L. (2016). Australian, Irish and Swedish women’s perceptions of what assisted them to breastfeed for six months: Exploratory design using critical incident technique. BMC Public Health.

[66] Corkery-Hayward M., Talaei M. (2024). Teleintervention’s effects on breastfeeding in low-income women in high income countries: A systematic review and meta-analysis. Int. Breastfeed. J..

[67] Sun Y., Gao Y., Zhu Z., Zhu L. (2023). Effect of online intervention mode on breastfeeding results: A systematic review and meta-analysis. Reprod. Health.

[68] Wong K.L., Tarrant M., Lok K.Y. (2015). Group versus Individual Professional Antenatal Breastfeeding Education for Extending Breastfeeding Duration and Exclusivity: A Systematic Review. J. Hum. Lact..

[69] Skouteris H., Nagle C., Fowler M., Kent B., Sahota P., Morris H. (2014). Interventions designed to promote exclusive breastfeeding in high-income countries: A systematic review. Breastfeed. Med..

[70] Zhao Z.H., Huang Y.Y., Qiao J., Huang W.P., Redding S.R., Wang R., Ouyang Y.Q. (2023). Co-Parenting Impact on Breastfeeding: Systematic Review and Meta-Analysis. Breastfeed. Med..

[71] Negin J., Coffman J., Vizintin P., Raynes-Greenow C. (2016). The influence of grandmothers on breastfeeding rates: A systematic review. BMC Pregnancy Childbirth.

[72] Awelewa T., Murra A., Story W.T. (2025). Developing a Framework for Culturally Sensitive Breastfeeding Interventions: A Community Needs Assessment of Breastfeeding Experiences and Practices in a Black Immigrant Community. Nutrients.

[73] American Academy of Pediatrics (2022). Cultural Differences in Infant Feeding. American Academy of Pediatrics. https://www.aap.org/en/patient-care/newborn-and-infant-nutrition/cultural-differences-in-infant-feeding/?srsltid=AfmBOorgEpvBddb2rimI2AewwUdxm_s2_0c_ocQy61ktdL8sjZgm2hiA.

[74] Nan Y., Zhang J., Nisar A., Huo L., Yang L., Yin J., Wang D., Rahman A., Gao Y., Li X. (2020). Professional support during the postpartum period: Primiparous mothers’ views on professional services and their expectations, and barriers to utilizing professional help. BMC Pregnancy Childbirth.

