# Supplementary material for: Interventions Aiming to Improve Breastfeeding Duration Among Primiparous Women: A Scoping Review
Source: Pediatr Rep. 2026 Mar 3;18(2):35. doi: 10.3390/pediatric18020035 (PMC13010726; doi:10.3390/pediatric18020035)
Supplement: Supplementary file 1 [file pediatrrep-18-00035-s001.zip › pediatrrep-4028824 supplement material.pdf]

Table S1. Final MEDLINE Search Strategy

1.	postnatal care/
2.	health education/
3.	health promotion/
4.	exp social support/
5.	consultant/
6.	follow up/
7.	early intervention, educational/
8.	exp maternal health services/
9.	prenatal care/
10.	(intervention* or training or consultation* or consultant* or "social support" or "follow-up stud*" or "maternal health service*" or "International lactation consultant association").ab,kf,kw,ti.
11.	(health adj2 (promotion or campaign* or education* or care)).ab,kf,kw,ti.
12.	((postpartum or postnatal or prenatal or "head start" or social or "long term" or wellness) adj2 (care or program? or service*)).ab,kf,kw,ti.
13.	1 or 2 or 3 or 4 or 5 or 6 or 7 or 8 or 9 or 10 or 11 or 12
14.	exp breast feeding/
15.	lactation/
16.	(breastfeeding or "breast feeding" or "breast fed" or "breastfed" or "chest feeding" or chestfeeding or pumping or "breast milk expressing" or "breastmilk expressing" or "breast milk expression" or "breastmilk expression" or "human milk" or lactation or lactating or "milk sharing" or "infant feeding").ab,kf,kw,ti.
17.	14 or 15 or 16
18.	mother/
19.	mother*.ab,kf,kw,ti.
20.	((female or wom#n or pregnan*) adj4 ("first-time" or primiparous or nulliparous)).ab,kf,kw,ti.
21.	18 or 19 or 20
22.	13 and 17 and 21
23.	limit 22 to (yr="2013 -Current" and (english or french))

Table S2. Risk of bias domains: author judgments about each RCT included in the scoping review [1]

	Randomisation process	Risk of bias arising from the timing of identification or recruitment of participants	Deviations from intended interventions	Missing outcome data	Measurement of the outcome	Selection of the reported result	Overall bias
Abbass-dick 2015	-	-	-	-	-	-	-
Abbass-dick 2020	-	-	-	-	-	-	-
Abbott 2019	-	-	-	+	-	-	+
Ansari 2014	-	-	-	-	-	-	-
Bunik 2022	-	-	-	+	-	-	+
Cangol 2017	-	-	-	-	-	-	-
Chegeni 2022	-	-	-	-	-	-	-
Chehreh 2021	-	-	-	+	-	-	+
Demirci 2022	-	-	++	+	-	-	++
Forster 2019	-	-	-	-	-	-	-
Gonzalez-Darias 2020	-	-	+	+	-	-	+
Gu 2016	-	-	-	+	-		+
Hans 2018	-	-	-	-	-	-	-
Hermanson 2020	-	-	+	+	-	-	+
Hoyt-Austin 2023	-	-	-	++	-	-	++
Huynh 2016	-	-	-	-	-	-	-
Lewkowitz 2020	-	-	-	++	-	-	++
Panahi 2022	-	-	-	-	-	-	-
Prasitwattanaseree 2019	-	-	-	-	-	-	-
Puharic 2020	-	-	-	-	-	-	-
Shariat 2016	-	-	-	++	++	-	++
Taheri 2022	-	-	-	-	-	-	-
Tseng 2020	-	-	-	-	-	-	-
Wong 2014	-	-	-	-	-	-	-
Yi 2016	-	-	-	-	-	-	-
Yilmaz 2021	-	-	-	-	-	-	-
Yin 2021	-	-	-	+	-	-	+
Zhang 2021	++	-	+	-	-	-	++
Zhao 2021	-	-	-	-	-	-	-

[1] **-** Low risk of bias; **+** moderate risk of bias (some concerns); **++** High risk of bias.

Table S3. Risk of bias: author judgments about non-randomized studies of interventions included in the scoping review [1]

	Bias due to confounding	Bias in selection of participants into the study	Bias in classification of interventions	Bias due to deviations from intended interventions	Bias due to missing data	Bias in measurement of outcomes	Bias in selection of the reported result	Overall bias
Jiang 2014	+	+	-	-	-	-	-	+
Ke 2018	+	+	-	-	-	-	-	+
Meedya 2014	+	+	-	-	-	-	-	+
Naroee 2020	++	+	-	-	-	-	-	++
Rosuzeita 2018	+	-	-	-	-	-	-	+
Su 2016	++	-	-	-	-	-	-	++

[1] **-** Low risk of bias; **+** moderate risk of bias (some concerns); **++** High risk of bias.
